# Crystal structure of 2-methyl­sulfanyl-1-(thio­morpholin-4-yl)­ethanone

**DOI:** 10.1107/S2056989015015418

**Published:** 2015-08-22

**Authors:** Gihaeng Kang, Jineun Kim, Eunjin Kwon, Tae Ho Kim

**Affiliations:** a Department of Chemistry and Research Institute of Natural Sciences, Gyeongsang, National University, Jinju 52828, Republic of Korea

**Keywords:** crystal structure, thio­morpholine, hydrogen bonding

## Abstract

In the title compound, C_7_H_13_NOS_2_, the thio­morpholine ring adopts a chair conformation and the bond-angle sum at the N atom is 360°. The dihedral angle between the amide group and the thio­morpholine ring (all atoms) is 36.48 (12)°. In the crystal, C—H⋯O and C—H⋯S hydrogen bonds link adjacent mol­ecules, forming two-dimensional networks extending parellel to the (011) plane.

## Related literature   

For further information on the synthesis, see: Kim *et al.* (2008 ▸). For related crystal structures, see: Kim *et al.* (2006 ▸); Ujam *et al.* (2010 ▸).
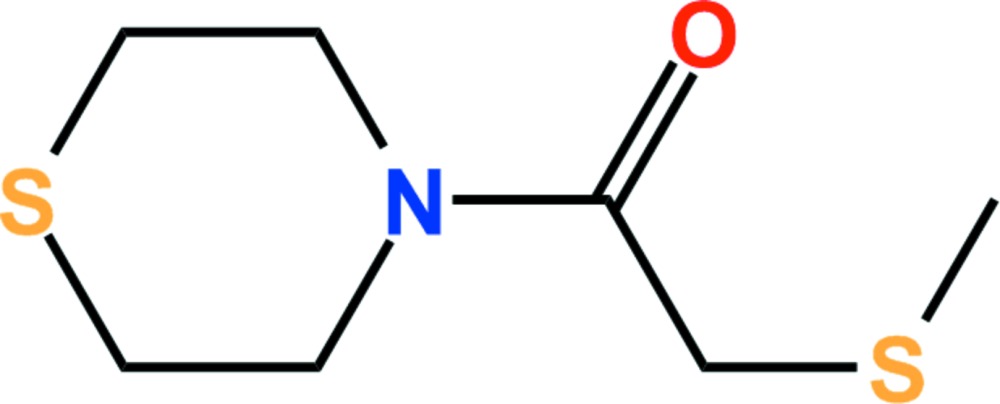


## Experimental   

### Crystal data  

C_7_H_13_NOS_2_
*M* *_r_* = 191.30
Monoclinic, 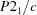

*a* = 15.0461 (15) Å
*b* = 6.1525 (6) Å
*c* = 10.4751 (10) Å
β = 107.581 (6)°
*V* = 924.40 (16) Å^3^
*Z* = 4
Mo *K*α radiation
μ = 0.52 mm^−1^
*T* = 173 K
0.23 × 0.18 × 0.08 mm

### Data collection  

Bruker APEXII CCD diffractometer
Absorption correction: multi-scan (*SADABS*; Bruker, 2013 ▸) *T*_min_ = 0.890, *T*_max_ = 0.959
8512 measured reflections
2111 independent reflections
1865 reflections with *I* > 2σ(*I*)
*R*_int_ = 0.026

### Refinement  

*R*[*F* ^2^ > 2σ(*F* ^2^)] = 0.029
*wR*(*F* ^2^) = 0.078
*S* = 1.05
2111 reflections
101 parameters
H-atom parameters constrained
Δρ_max_ = 0.22 e Å^−3^
Δρ_min_ = −0.27 e Å^−3^

### 

Data collection: *APEX2* (Bruker, 2013 ▸); cell refinement: *SAINT* (Bruker, 2013 ▸); data reduction: *SAINT*; program(s) used to solve structure: *SHELXS97* (Sheldrick, 2008 ▸); program(s) used to refine structure: *SHELXL2013* (Sheldrick, 2015 ▸); molecular graphics: *DIAMOND* (Brandenburg, 2010 ▸); software used to prepare material for publication: *SHELXTL* (Sheldrick, 2008 ▸).

## Supplementary Material

Crystal structure: contains datablock(s) global, I. DOI: 10.1107/S2056989015015418/hb7480sup1.cif

Structure factors: contains datablock(s) I. DOI: 10.1107/S2056989015015418/hb7480Isup2.hkl

Supporting information file. DOI: 10.1107/S2056989015015418/hb7480Isup3.cml

**Fig. 1.**
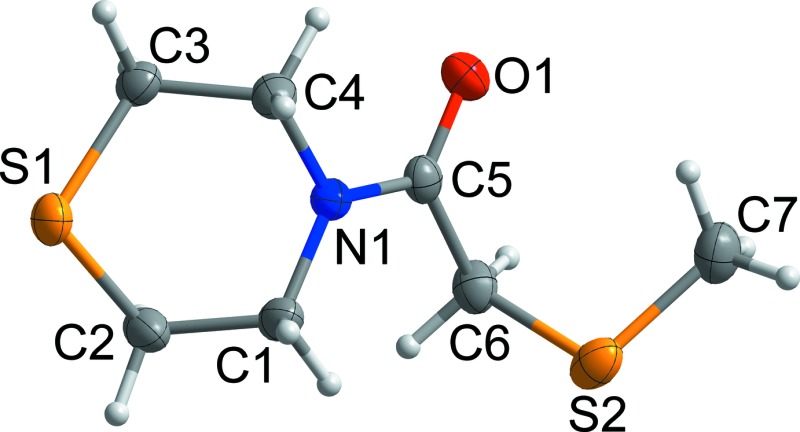
The asymmetric unit of the title compound with displacement ellipsoids drawn at the 50% probability level.

**Fig. 2.**
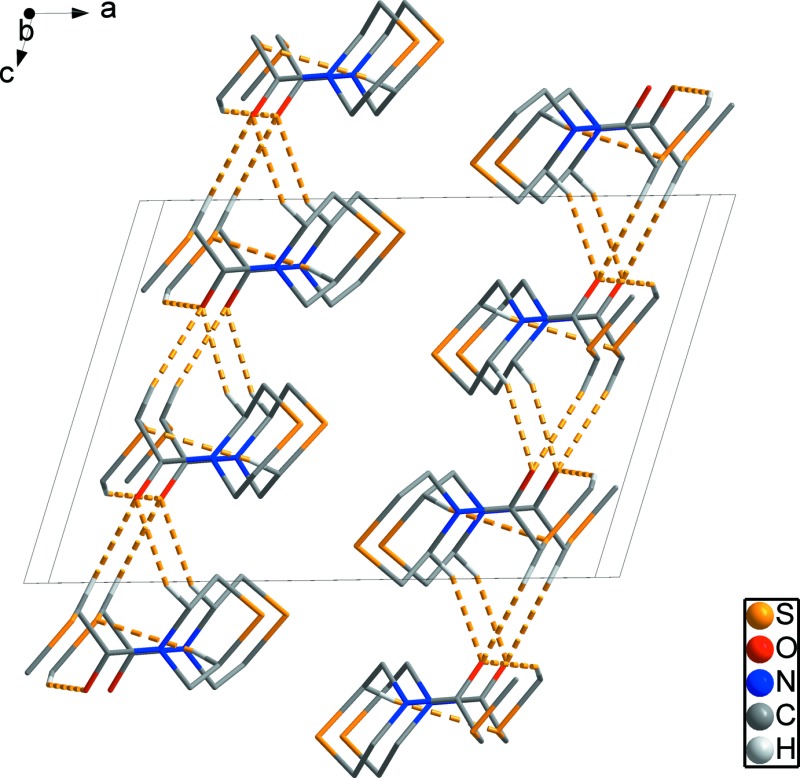
Crystal packing viewed along the b axis. The intermolecular C—H···O and C—H···S hydrogen bonds are shown as dashed lines.

. DOI: 10.1107/S2056989015015418/hb7480fig1.tif
The asymmetric unit of the title compound with displacement ellipsoids drawn at the 50% probability level.

b . DOI: 10.1107/S2056989015015418/hb7480fig2.tif
Crystal packing viewed along the *b* axis. The inter­molecular C—H⋯O and C—H⋯S hydrogen bonds are shown as dashed lines.

CCDC reference: 1419333

Additional supporting information:  crystallographic information; 3D view; checkCIF report

## Figures and Tables

**Table 1:** Hydrogen-bond geometry (, )

*D*H*A*	*D*H	H*A*	*D* *A*	*D*H*A*
C1H1*B*O1^i^	0.99	2.46	3.3490(19)	150
C6H6*B*O1^i^	0.99	2.59	3.4427(18)	144
C7H7*B*O1^ii^	0.98	2.45	3.3237(19)	148
C3H3*A*S2^iii^	0.99	2.88	3.8201(15)	159

Symmetry codes: (i) 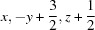; (ii) 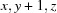; (iii) 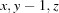.

## supplementary crystallographic information

### S1. Comment

..

### S2. Experimental

Thionyl chloride (2.38 g, 20.0 mmol) was added dropwise to 2-methylthioacetic acid (2.12 g, 20.0 mmol) in the pesence of triethylamine (2.02 g, 20.0 mmol) in chloroform. The mixture was refluxed for 2 h and cooled down to room temperature. Then, thiomorpholine (2.38 g, 20.0 mmol) and triethylamine (2.02 g, 20.0 mmol) in chloroform were added dropwise to the resulting acid chloride solution, cooled by salt and ice water. The solution was stirred for 2 h, and then water was added. Organic layer was collected and water layer was extracted with chloroform. The combined organic layers dried with anhydrous sodium sulfate were evaporated to give crude oil. Column chromatography (silica gel, ethyl acetate/hexane = 20/80 (*v*/*v*), *R*_f_ 0.1) gave pure title compound (3.42 g, 89%) (Kim *et al.*, 2008). Slow evaporation of a solution in acetone/ethyl acetate gave colourless blocks.

### S3. Refinement

All H-atoms were positioned geometrically and refined using a riding model with d(C—H) = 0.99 Å, *U*_iso_ = 1.2*U*_eq_(C) for CH_2_ groups and d(C—H) = 0.98 Å, *U*_iso_ = 1.5*U*_eq_(C) for CH_3_ group.

### Crystal data

**Table:**

C_7_H_13_NOS_2_	*F*(000) = 408
*M**_r_* = 191.30	*D*_x_ = 1.375 Mg m^−^^3^
Monoclinic, *P*2_1_/*c*	Mo *K*α radiation, λ = 0.71073 Å
*a* = 15.0461 (15) Å	Cell parameters from 4186 reflections
*b* = 6.1525 (6) Å	θ = 2.8–27.5°
*c* = 10.4751 (10) Å	µ = 0.52 mm^−^^1^
β = 107.581 (6)°	*T* = 173 K
*V* = 924.40 (16) Å^3^	Block, colourless
*Z* = 4	0.23 × 0.18 × 0.08 mm

### Data collection

**Table:**

Bruker APEXII CCD diffractometer	1865 reflections with *I* > 2σ(*I*)
φ and ω scans	*R*_int_ = 0.026
Absorption correction: multi-scan (*SADABS*; Bruker, 2013)	θ_max_ = 27.5°, θ_min_ = 2.8°
*T*_min_ = 0.890, *T*_max_ = 0.959	*h* = −19→19
8512 measured reflections	*k* = −7→7
2111 independent reflections	*l* = −13→13

### Refinement

**Table:**

Refinement on *F*^2^	0 restraints
Least-squares matrix: full	Hydrogen site location: inferred from neighbouring sites
*R*[*F*^2^ > 2σ(*F*^2^)] = 0.029	H-atom parameters constrained
*wR*(*F*^2^) = 0.078	*w* = 1/[σ^2^(*F*_o_^2^) + (0.0406*P*)^2^ + 0.2667*P*] where *P* = (*F*_o_^2^ + 2*F*_c_^2^)/3
*S* = 1.05	(Δ/σ)_max_ = 0.001
2111 reflections	Δρ_max_ = 0.22 e Å^−^^3^
101 parameters	Δρ_min_ = −0.27 e Å^−^^3^

### Special details

**Table:**

Geometry. All e.s.d.'s (except the e.s.d. in the dihedral angle between two l.s. planes) are estimated using the full covariance matrix. The cell e.s.d.'s are taken into account individually in the estimation of e.s.d.'s in distances, angles and torsion angles; correlations between e.s.d.'s in cell parameters are only used when they are defined by crystal symmetry. An approximate (isotropic) treatment of cell e.s.d.'s is used for estimating e.s.d.'s involving l.s. planes.

### Fractional atomic coordinates and isotropic or equivalent isotropic displacement parameters (Å^2^)

**Table:**

	*x*	*y*	*z*	*U*_iso_*/*U*_eq_	
S1	0.57059 (2)	0.35556 (6)	0.41078 (4)	0.03060 (12)	
S2	0.87043 (3)	1.06954 (6)	0.40092 (4)	0.03528 (13)	
O1	0.82963 (8)	0.59419 (17)	0.21785 (11)	0.0333 (2)	
N1	0.72025 (8)	0.62668 (19)	0.32412 (12)	0.0264 (3)	
C1	0.68637 (10)	0.7095 (2)	0.43112 (15)	0.0315 (3)	
H1A	0.6300	0.7989	0.3920	0.038*	
H1B	0.7346	0.8037	0.4909	0.038*	
C2	0.66298 (11)	0.5254 (3)	0.51228 (15)	0.0325 (3)	
H2A	0.6437	0.5870	0.5871	0.039*	
H2B	0.7193	0.4357	0.5510	0.039*	
C3	0.62268 (10)	0.2963 (2)	0.28028 (15)	0.0280 (3)	
H3A	0.6779	0.2023	0.3172	0.034*	
H3B	0.5774	0.2148	0.2078	0.034*	
C4	0.65213 (10)	0.5000 (2)	0.22214 (14)	0.0296 (3)	
H4A	0.6795	0.4586	0.1507	0.036*	
H4B	0.5965	0.5909	0.1812	0.036*	
C5	0.80749 (9)	0.6585 (2)	0.31488 (14)	0.0243 (3)	
C6	0.87815 (10)	0.7787 (2)	0.42581 (15)	0.0297 (3)	
H6A	0.9416	0.7294	0.4300	0.036*	
H6B	0.8679	0.7434	0.5125	0.036*	
C7	0.91981 (12)	1.0968 (3)	0.26585 (18)	0.0385 (4)	
H7A	0.9862	1.0593	0.2975	0.058*	
H7B	0.9127	1.2471	0.2334	0.058*	
H7C	0.8876	0.9988	0.1928	0.058*	

### Atomic displacement parameters (Å^2^)

**Table:**

	*U*^11^	*U*^22^	*U*^33^	*U*^12^	*U*^13^	*U*^23^
S1	0.0255 (2)	0.0330 (2)	0.0357 (2)	−0.00719 (13)	0.01283 (16)	−0.00026 (15)
S2	0.0262 (2)	0.0305 (2)	0.0515 (3)	−0.00455 (14)	0.01529 (18)	−0.01097 (16)
O1	0.0347 (6)	0.0363 (5)	0.0344 (6)	−0.0013 (4)	0.0187 (5)	−0.0022 (4)
N1	0.0262 (6)	0.0290 (6)	0.0263 (6)	−0.0077 (5)	0.0115 (5)	−0.0052 (5)
C1	0.0313 (8)	0.0309 (7)	0.0369 (8)	−0.0085 (6)	0.0173 (7)	−0.0101 (6)
C2	0.0323 (8)	0.0403 (8)	0.0279 (8)	−0.0093 (6)	0.0134 (6)	−0.0064 (6)
C3	0.0251 (7)	0.0268 (7)	0.0325 (8)	−0.0050 (5)	0.0092 (6)	−0.0058 (6)
C4	0.0288 (7)	0.0341 (7)	0.0248 (7)	−0.0081 (6)	0.0065 (6)	−0.0028 (6)
C5	0.0251 (7)	0.0212 (6)	0.0278 (7)	0.0011 (5)	0.0099 (6)	0.0051 (5)
C6	0.0229 (7)	0.0349 (7)	0.0300 (8)	−0.0034 (6)	0.0059 (6)	0.0037 (6)
C7	0.0374 (9)	0.0309 (8)	0.0490 (10)	−0.0021 (6)	0.0157 (8)	0.0058 (7)

### Geometric parameters (Å, º)

**Table:**

S1—C2	1.8061 (15)	C2—H2B	0.9900
S1—C3	1.8065 (14)	C3—C4	1.517 (2)
S2—C7	1.7935 (17)	C3—H3A	0.9900
S2—C6	1.8067 (16)	C3—H3B	0.9900
O1—C5	1.2267 (17)	C4—H4A	0.9900
N1—C5	1.3592 (17)	C4—H4B	0.9900
N1—C1	1.4563 (17)	C5—C6	1.511 (2)
N1—C4	1.4622 (18)	C6—H6A	0.9900
C1—C2	1.520 (2)	C6—H6B	0.9900
C1—H1A	0.9900	C7—H7A	0.9800
C1—H1B	0.9900	C7—H7B	0.9800
C2—H2A	0.9900	C7—H7C	0.9800
			
C2—S1—C3	97.45 (6)	H3A—C3—H3B	107.8
C7—S2—C6	100.51 (7)	N1—C4—C3	111.87 (12)
C5—N1—C1	125.07 (12)	N1—C4—H4A	109.2
C5—N1—C4	120.24 (11)	C3—C4—H4A	109.2
C1—N1—C4	114.68 (11)	N1—C4—H4B	109.2
N1—C1—C2	111.34 (12)	C3—C4—H4B	109.2
N1—C1—H1A	109.4	H4A—C4—H4B	107.9
C2—C1—H1A	109.4	O1—C5—N1	121.49 (13)
N1—C1—H1B	109.4	O1—C5—C6	119.42 (12)
C2—C1—H1B	109.4	N1—C5—C6	119.09 (12)
H1A—C1—H1B	108.0	C5—C6—S2	111.99 (10)
C1—C2—S1	111.64 (11)	C5—C6—H6A	109.2
C1—C2—H2A	109.3	S2—C6—H6A	109.2
S1—C2—H2A	109.3	C5—C6—H6B	109.2
C1—C2—H2B	109.3	S2—C6—H6B	109.2
S1—C2—H2B	109.3	H6A—C6—H6B	107.9
H2A—C2—H2B	108.0	S2—C7—H7A	109.5
C4—C3—S1	112.52 (10)	S2—C7—H7B	109.5
C4—C3—H3A	109.1	H7A—C7—H7B	109.5
S1—C3—H3A	109.1	S2—C7—H7C	109.5
C4—C3—H3B	109.1	H7A—C7—H7C	109.5
S1—C3—H3B	109.1	H7B—C7—H7C	109.5
			
C5—N1—C1—C2	−115.63 (15)	C1—N1—C5—O1	−175.84 (13)
C4—N1—C1—C2	64.06 (16)	C4—N1—C5—O1	4.5 (2)
N1—C1—C2—S1	−62.22 (15)	C1—N1—C5—C6	3.2 (2)
C3—S1—C2—C1	53.57 (12)	C4—N1—C5—C6	−176.45 (12)
C2—S1—C3—C4	−52.50 (12)	O1—C5—C6—S2	92.67 (14)
C5—N1—C4—C3	117.12 (14)	N1—C5—C6—S2	−86.42 (13)
C1—N1—C4—C3	−62.59 (16)	C7—S2—C6—C5	−73.01 (11)
S1—C3—C4—N1	59.54 (14)		

### Hydrogen-bond geometry (Å, º)

**Table:**

*D*—H···*A*	*D*—H	H···*A*	*D*···*A*	*D*—H···*A*
C1—H1*B*···O1^i^	0.99	2.46	3.3490 (19)	150
C6—H6*B*···O1^i^	0.99	2.59	3.4427 (18)	144
C7—H7*B*···O1^ii^	0.98	2.45	3.3237 (19)	148
C3—H3*A*···S2^iii^	0.99	2.88	3.8201 (15)	159

Symmetry codes: (i) *x*, −*y*+3/2, *z*+1/2; (ii) *x*, *y*+1, *z*; (iii) *x*, *y*−1, *z*.
