# Supplementary figures and images for: Crystal structure of 2-methyl­sulfanyl-1-(thio­morpholin-4-yl)­ethanone
Source: Acta Crystallogr E Crystallogr Commun. 2015 Aug 22;71(Pt 9):o679. doi: 10.1107/S2056989015015418 (PMC4555416; doi:10.1107/S2056989015015418)

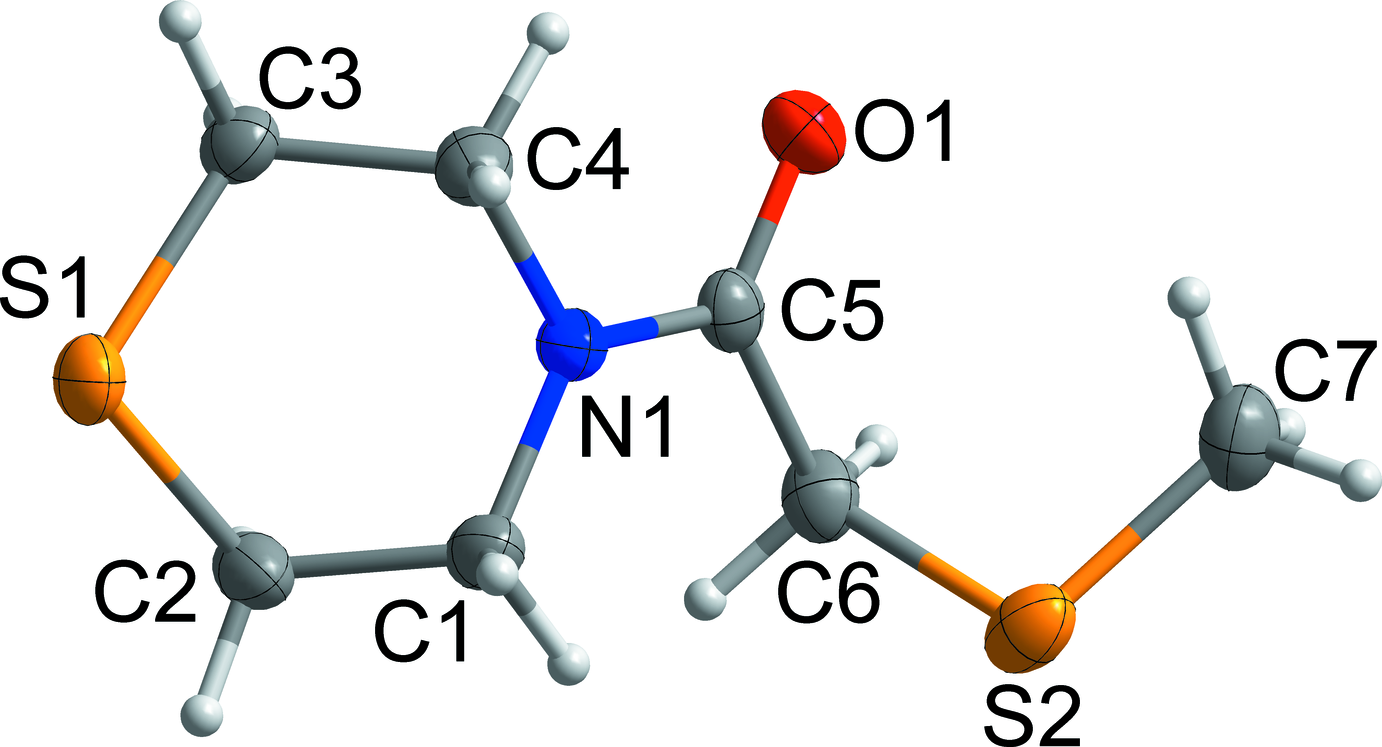

Supplement: Supplementary file 4 [file e-71-0o679-fig1.tif]

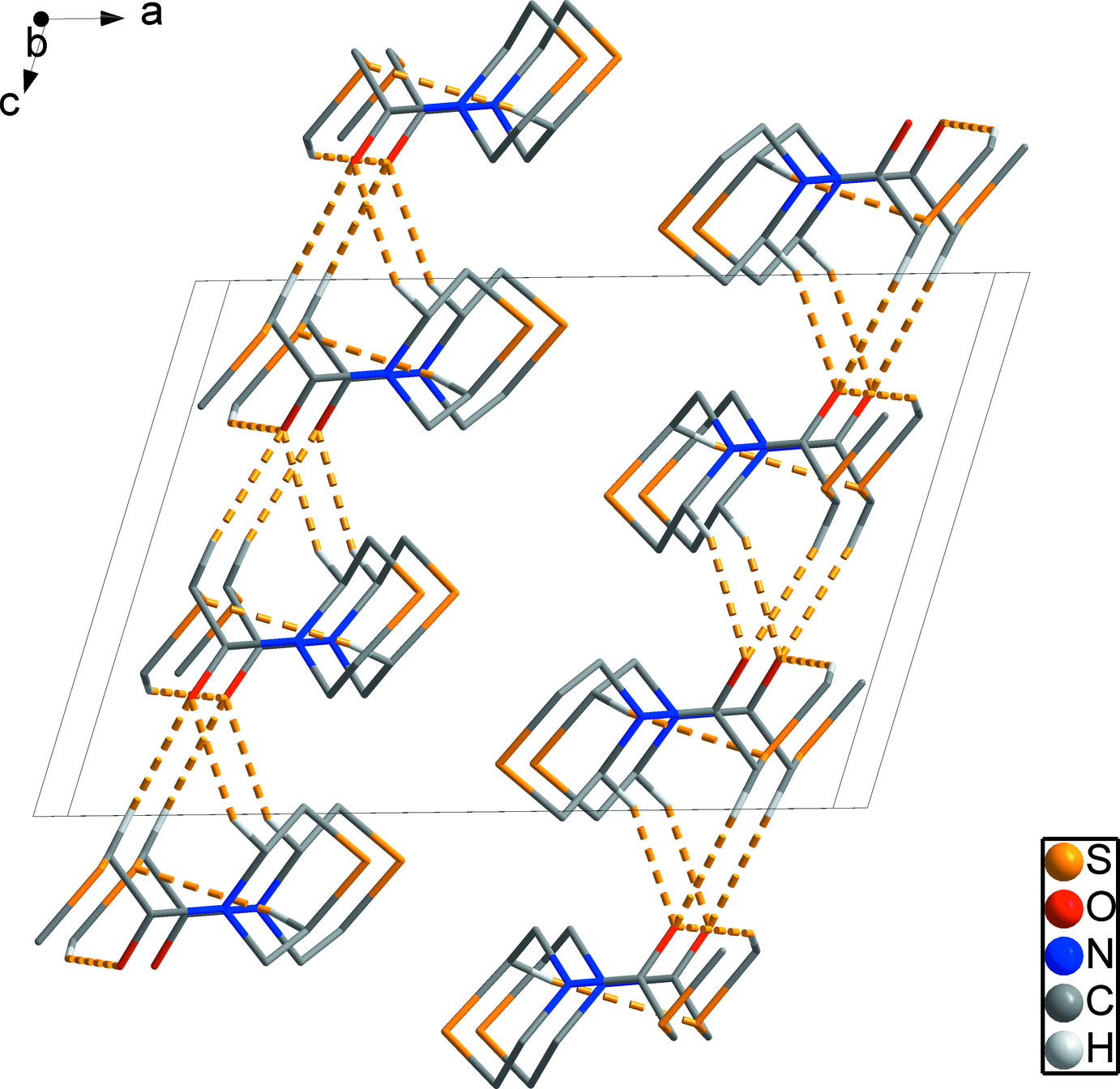

Supplement: Supplementary file 5 [file e-71-0o679-fig2.tif]
